# Current knowledge of thrombocytopenia in sepsis and COVID-19

**DOI:** 10.3389/fimmu.2023.1213510

**Published:** 2023-09-28

**Authors:** Junjie Cheng, Hanhai Zeng, Huaijun Chen, Linfeng Fan, Chaoran Xu, Huaping Huang, Tianchi Tang, Min Li

**Affiliations:** ^1^ Intensive Care Unit, The Fourth Affiliated Hospital, School of Medicine, Zhejiang University, Yiwu, China; ^2^ Department of Neurosurgery, The Second Affiliated Hospital, School of Medicine, Zhejiang University, Hangzhou, China

**Keywords:** platelet count, sepsis, thrombocytopenia, COVID-19, platelet

## Abstract

Thrombocytopenia, characterized by a decrease in platelet count, is commonly observed in sepsis and COVID-19. In sepsis, thrombocytopenia can result from various mechanisms, including impaired platelet production in the bone marrow, accelerated platelet destruction due to increased inflammation, sequestration of platelets in the spleen, immune-mediated platelet destruction, or dysregulated host responses. Similarly, thrombocytopenia has been reported in COVID-19 patients, but the immune-related mechanisms underlying this association remain unclear. Notably, interventions targeting thrombocytopenia have shown potential for improving outcomes in both sepsis and COVID-19 patients. Understanding these mechanisms is crucial for developing effective treatments.

## 1 Introduction

Thrombocytopenia refers to a condition characterized by abnormally low levels of blood platelets—a vital component of the body’s clotting system. It can be triggered by various factors such as medications, infections, autoimmune diseases, and other health conditions (1). Thrombocytopenia can lead to symptoms like excessive bruising and bleeding and may result in severe complications such as an increased risk of stroke, heart attack, or death (1). Investigating the causes and exploring effective treatments for thrombocytopenia are essential for improving patient prognosis and quality of life.

Sepsis is a life-threatening condition characterized by excessive inflammation caused by infection. It has seen a significant increase in incidence—8.7% over recent decades—due to the body’s exaggerated response to infections (2). Coagulation disorders play a major role in sepsis-related mortality ranging from mild thrombocytopenia to fatal conditions like disseminated intravascular coagulation (DIC) (3). Platelets are crucial in sepsis, and thrombocytopenia serves as both a prognostic marker and an independent predictor of worse outcomes (4, 5). Notably, thrombocytopenia during sepsis is associated with increased overall 30-day mortality (6). Furthermore, platelets have the potential to be therapeutic targets and modulators of sepsis (7), highlighting the significance of understanding thrombocytopenia in sepsis.

In recent years, the COVID-19 pandemic has profoundly impacted healthcare systems and individuals’ health. Extensive research has shown that SARS-CoV-2—the virus causing COVID-19—affects various tissues and organs beyond the respiratory system. Studies have revealed that SARS-CoV-2 can directly or indirectly affect blood cells such as hematopoietic stem cells, megakaryocytes, and platelets (8, 9). Approximately one-quarter of COVID-19 patients develop thrombocytopenia Ashwell-Morell receptor, particularly within the first week after admission (8, 9). Thrombocytopenia in ICU-admitted COVID-19 patients has been associated with a significantly worse prognosis for severe cases (10), and even patients with normal platelet count at admission but developing thrombocytopenia during ICU stay exhibited lower survival rates compared to those without thrombocytopenia (11). Despite these observations, the mechanisms underlying COVID-19-associated thrombocytopenia remain poorly understood.

Therefore, this review aims to summarize the current knowledge regarding thrombocytopenia in sepsis and its association with COVID-19.

## 2 Mechanisms of thrombocytopenia in sepsis

Several complex mechanisms contribute to thrombocytopenia during sepsis. Dysregulated host responses, interactions with platelet receptors and complexes, and immune-mediated thrombocytopenia are among the identified mechanisms (12–14). However, the precise mechanisms behind these phenomena require further elucidation. Understanding these mechanisms is crucial for developing appropriate treatment strategies (15, 16).

### 2.1 Dysregulated host response

Thrombocytopenia can exacerbate the disturbed host response observed in sepsis, as evidenced by animal models and functional data (17). Disease severity strongly influences host response biomarkers in sepsis (12, 18). Interleukin mediators play a role in the host response, with elevated plasma levels of IL-6, IL-8, and IL-10 indicating cytokine network activation. Additionally, vascular endothelial activation (elevated soluble E-selectin and soluble ICAM-1) and compromised vascular integrity are observed. Coagulation abnormalities—prolonged aPTT and PT, increased plasma D-dimer levels, decreased antithrombin and protein C levels—contribute to thrombocytopenia in sepsis (12, 17, 18).

### 2.2 The interaction between platelet receptors and complexes

Platelet-receptor glycoprotein Ibalpha (GpIbalpha), part of the GP Ib-IX-V complex, and plasma von Willebrand factor (VWF) proteins play a role in hemostasis and the process of platelet attachment to the vascular endothelium (19). In septic shock, platelet inhibition prevents clotting, preserves endothelial function, and reduces tissue damage, potentially leading to improved outcomes (20).

The Toll-like receptors (TLRs) of the innate immune system recognize molecules of microbial origin by interacting with their transmembrane domains (21). TLR2, TLR4, and TLR9 are expressed on the surface of platelets. During sepsis, TLRs are activated, with TLR4 being involved in endotoxemia by recognizing lipopolysaccharide (LPS) proteins. Furthermore, LPS-induced thrombocytopenia is reduced because the expression of TLR4 is significantly decreased in activated platelets (13, 14). Studies have shown that the increase in cGMP in an LPS-induced TLR knockout model is also mediated through the TLR4 pathway and inhibited by anti-TLR4 blocking antibodies. TLR4 interacts with LPS to promote platelet secretion and enhance platelet aggregation (22).

**Figure 1:**
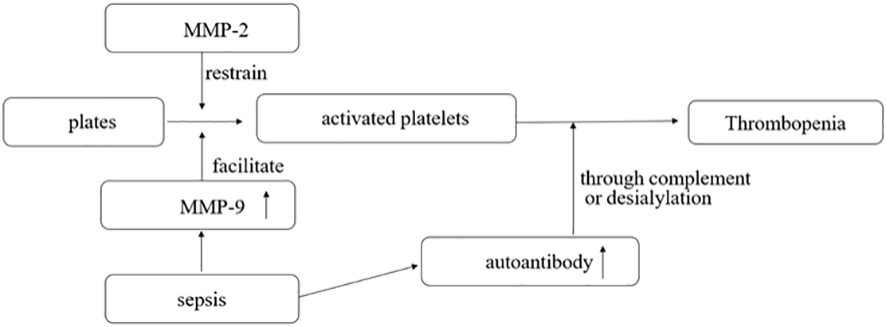
MMP-2 is a platelet agonist, and MMP-9 is a platelet activation inhibitor. In sepsis, the pathogen interacts with the body to produce a large number of antibodies, some of which can destroy activated platelets through complement or desialylation.

Matrix metalloproteinases (MMPs) are enzymes that modulate extracellular matrix recycling. MMP-2 is a platelet agonist, while MMP-9 is a platelet activation inhibitor (**Figure 1**). Limited evidence suggests that toll-like receptor 4 (TLR-4) formation and platelet-leukocyte aggregates (PLA) may be associated with the development of sepsis-associated thrombocytopenia. However, current studies have found no difference in levels of MMP-2, MMP-9, and TLR-4 between donors with non-thrombotic and thrombotic sepsis. PLA formation is also increased in patients with thrombocytopenia. MMP-9 in platelets was detected using flow cytometry, gelatin enzyme spectrometry, and ELISA (23). Platelet consumption into the plasma may be the cause of thrombocytopenia in septic shock. The expression of MMP-9 in platelets increases during septic shock, suggesting that MMP-9 could be a potential therapeutic target for thrombocytopenia in sepsis (23).

### 2.3 Immune-mediated thrombocytopenia

**Figure 2:**
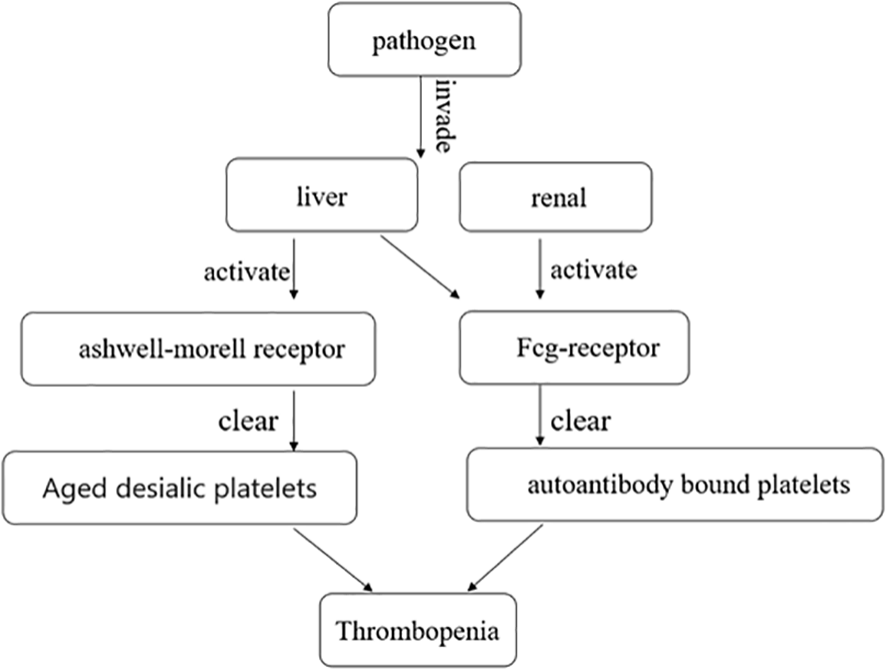
After the pathogen invades the liver and kidney, it clears aged desialic platelets and autoantibody-bound platelets by activating the Ashwell-Morell receptor and Fcg-receptor, respectively, causing thrombocytopenia.

The mechanism of immune-mediated thrombocytopenia is complex and still under exploration. The classical explanation is that platelets bound to autoantibodies on their surfaces are destroyed in the spleen or liver through interaction with Fcγ receptors (24). Autoantibodies can mediate platelet destruction through complement activation or desialylation (25–27). Aged desialylated platelets are cleared by the liver Ashwell-Morell receptor (AMR) (28). Recent studies have shown that conditioning platelets with anti-GPIB antibodies activate platelets, leading to the translocation of neuraminidase-1 to the surface, which desialylated platelets for Fc-independent hepatic clearance via AMR in the liver (26) (**Figure 2**). Studies have demonstrated that abnormal T cells, including T-helper (Th) cell differentiation into Th1 and Th17 phenotypes and reduced regulatory T cells, contribute to varying degrees of platelet destruction (24). Additionally, CD8+ T cells, especially cytotoxic CD8+ T cells, cause thrombocytopenia through phagocytosis of splenic macrophages or dendritic cells. Activation of CD8+ T cells in the bone marrow can also lead to damage to megakaryocytes and inhibit platelet production (24). Liver macrophages clear sialic acid-free platelets through macrophage galactolectin (29). Whether macrophage clearance of platelet aggregates affects antibody-induced hepatocellular-mediated platelet clearance remains unclear. Further research is still needed to understand the mechanism of immune-mediated thrombocytopenia.

It has been demonstrated that platelet levels of CD63 (LIMP-1), CD62P (P-selectin), and CD31 (platelet-endothelial cell adhesion molecule) are increased, while CD36 (GP IV) levels are significantly decreased during septic shock (30, 31). This suggests that platelet activation is mediated by interactions between platelets and leukocytes, endothelium, and activated endothelium, with platelet adhesion and aggregation being facilitated by these surface antigens (31).

**Figure 3:**
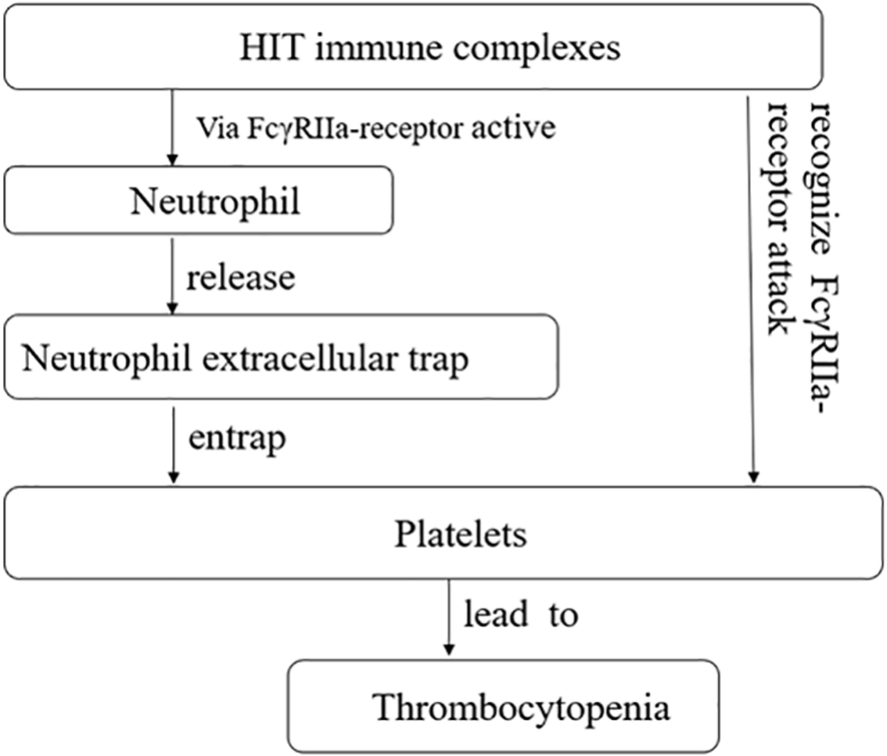
Heparin-induced thrombocytopenia (HIT) immune complexes via FcγRIIa-receptor active neutrophil, and further promotes the release of neutrophil extracellular trap, causing it to seize platelets, causing thrombocytopenia. HIT immune complexes attack plates through FcγRIIa-receptor, causing thrombocytopenia.

Additionally, heparin-induced thrombocytopenia (HIT) is typically an immune-mediated thrombocytopenia (32) and is mediated by IgG antibodies that have specificity for platelet factor 4 antigen complexes (33). The consensus view is that these immune complexes activate platelets through Fc gamma RIIa receptors, leading to thrombocytopenia and thrombosis (34). Neutrophil extracellular traps (NETs) are complex structures composed of DNA and various proteins, including histones, neutrophil elastase, myeloperoxidase, and antimicrobial proteins. They have been increasingly reported in patients with infections and thrombosis associated with autoimmune and non-immune diseases (35, 36). When activated neutrophils release NETs, they form a mesh-like structure that traps and prevents platelets from binding to their receptors, resulting in thrombocytopenia. HIT immune complexes also activate neutrophils and induce the formation of NETs through MMP (33) (**Figure 3**).

Additionally, activated platelet/neutrophil interactions mediated by P-selectin and PSGL-1 also induce NETosis (37). Research has shown that neutropenia eliminates thrombosis. Conversely, neutrophil reconstitution restores thrombus deposition and leads to thrombocytopenia (33). Furthermore, NETs can activate the complement system (38), leading to the generation of anaphylatoxins that can directly lyse platelets and further contribute to the development of thrombocytopenia.

Moreover, studies have found elevated levels of platelet-related IgG in sepsis with thrombocytopenia. Anti-platelet autoantibodies (anti-GP IIb/IX) have also been detected in a small number of patients, such as idiopathic thrombocytopenic purpura, suggesting a potential immune-related process for thrombocytopenia in sepsis (15). In mouse models of immune thrombocytopenia (ITP), monoclonal antibodies IgG, which bind to cell surface-associated antigens, prevent the development of immune thrombocytopenia (39, 40). It has been suggested that phagocytosis of reticuloendothelial cells in the bone marrow may also cause thrombocytopenia (41).

### 2.4 Disseminated intravascular coagulation

Disseminated intravascular coagulation (DIC) often occurs in patients with sepsis. It is mediated by pathogen-related molecules, leading to upregulated expression of tissue factors and inhibition of anticoagulation and fibrinolysis mechanisms (42). Platelet activation and white blood cell involvement are widely reported (43). The underlying disease process, such as trauma-induced mechanical endothelial injury, sepsis-related inflammation, obstetric complications, or cancer, results in excessive release of tissue factor, leading to overproduction of thrombin. Thrombin increases its production by converting soluble fibrinogen into insoluble fibrin chains and activating other thrombin and factors. Thrombin activates platelets as a key component of the primary hemostatic mechanism through the TF/VIIa axis and the involvement of Factor XIIa. Additionally, complement-mediated responses can affect lytic cells and/or bacterial pathogens through the release of damage-associated molecular patterns (DAMPs) or pathogen-associated molecular patterns (PAMPs) and other cellular components that promote blood coagulation (3).

Once a fibrinolytic clot begins to form, the fibrinolytic cascade is activated to counteract increased fibrin deposition and aggregation in microvessels. However, this fibrinolytic activity is impaired by anti-fibrinolytic components such as thrombin-activated fibrinolytic inhibitors (TAFI), plasminogen activator inhibitors (PAI-1), and other prethrombotic mediators (44, 45). Similarly, damage to the physiological anticoagulant pathway, including tissue factor pathway inhibitors (TFPI), antithrombin (AT), and activated protein C (APC), fails to inhibit the progressive procoagulant state to some extent. Currently, there is a bidirectional cross-talk between abnormal coagulation and inflammatory mediators, where inflammation effectively induces the coagulation cascade while abnormal coagulation profoundly alters and perpetuates inflammation (46, 47). In sepsis, major pro-inflammatory factors include IL-1, IL-6, TNF-α, elastase, and cathepsin (48). Recent research has elucidated the role of other thrombogenic inflammatory response factors such as NETs, extracellular vesicles, and shedding of endothelial calyx (49). DIC involves various mechanisms, including endothelial dysfunction and vascular endothelial injury, initiation of the coagulation pathway, platelet aggregation, dysfunction of the anticoagulation system, impaired fibrinolysis, activation of the complement system, upregulation of the inflammatory response, and many other factors that contribute to thrombocytopenia. Furthermore, it is assumed that all patients with sepsis have certain disorders of coagulation and clot activation regardless of obvious symptoms of disseminated intravascular coagulation (DIC). Moreover, DIC also correlates with disease severity (50).

### 2.5 Increased destruction of platelets

There are specific diseases that, when they cause sepsis, are also accompanied by thrombocytopenia. The mechanisms underlying this increased platelet destruction are not well understood. One example is hemolytic uremic syndrome, a thrombotic microvascular disease primarily caused by endothelial cell injury, leading to a series of syndromes (51). These factors collectively contribute to peripheral thrombocytopenia (51). Additionally, there are articles describing other causes of thrombocytopenia in sepsis, such as hypersplenism, bone marrow failure, heparin-induced thrombocytopenia (HIT), drug-induced thrombocytopenia (DIT), and blood dilution (42, 52, 53). Further research is needed to elucidate the mechanisms underlying these specific diseases.

## 3 Mechanisms of thrombocytopenia in COVID-19

**Figure 4:**
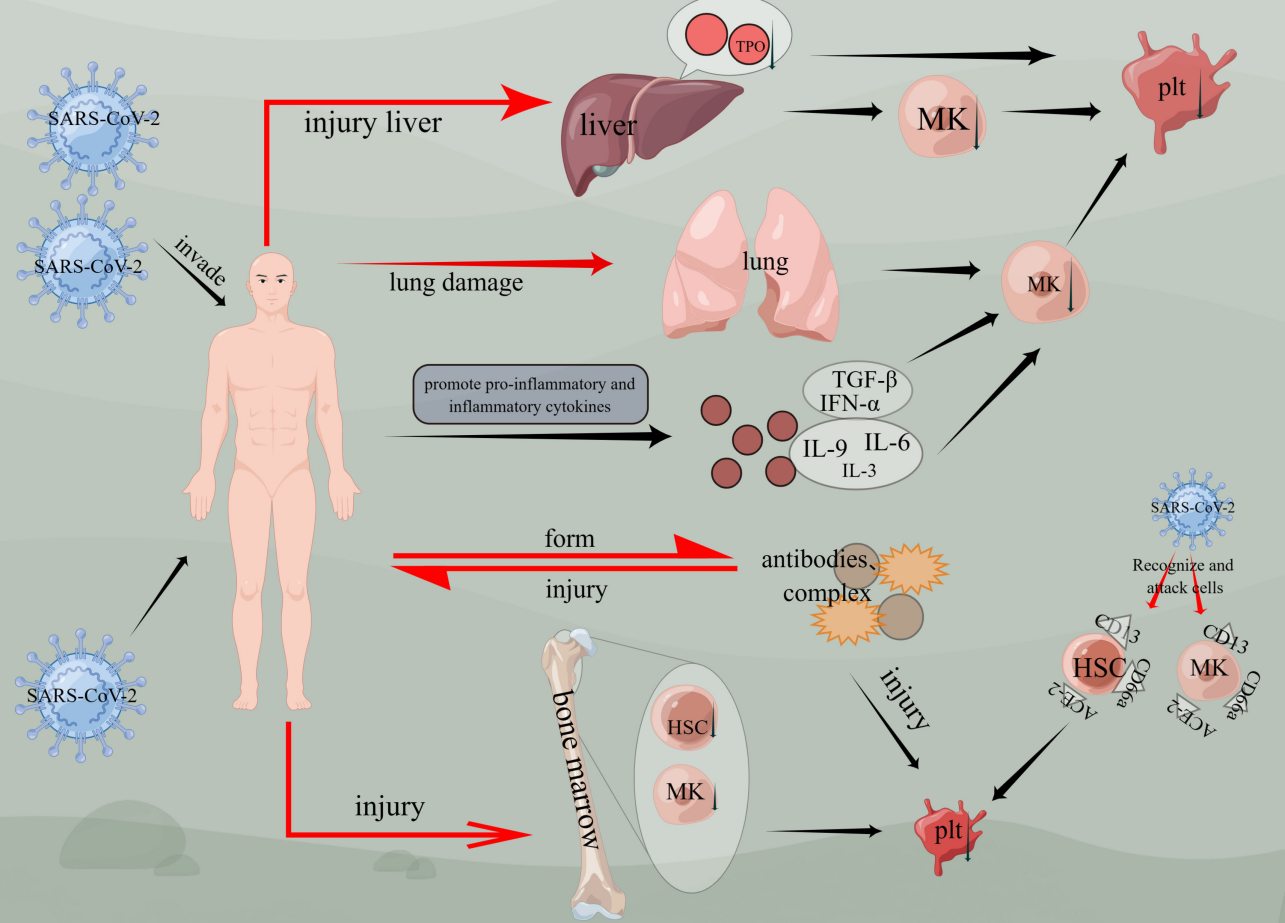
Mechanisms of thrombocytopenia caused by COVID-19. Viruses invade the body, cause liver and lung damage, and then damage megakaryocytes, causing thrombocytopenia. Viruses affect bone marrow hematopoietic function, affect hematopoietic stem cells and megakaryocyte generation, and cause thrombocytopenia. Thrombocytopenia is induced by CD13, ACE-2, and CD66a on hematopoietic stem cells or megakaryocytes. Inflammatory factors such as TGF-β, IFN-α, IL-3, IL-6, and IL-9 are activated, thus affecting the generation of megakaryocytes and the production of platelets. The virus enters the body and forms antibodies or compounds, and damages the body again. (figure by figdraw).

Based on previous studies, thrombocytopenia has been identified as one of the most common symptoms of COVID-19 (54). Furthermore, it has been observed that thrombocytopenia is associated with a threefold increased risk for severe COVID-19 and an elevated risk of mortality (55). Although the exact mechanisms of thrombocytopenia in COVID-19 are still being researched, several potential causes have been suggested. One prominent cause is the cytokine storm, which occurs when the immune system produces an excessive amount of cytokines that can destroy platelets and contribute to thrombocytopenia. Additionally, other causes include direct viral-induced cytopenias and immune-mediated destruction of platelets (56–58) (**Figure 4**).

### 3.1 Thrombocytopenia by affecting platelet production in COVID-19

Platelets are produced from megakaryocytes (MK), which derive from hematopoietic stem cells (59, 60). Hematopoietic tissue expresses ACE2 receptors, which are utilized by SARS-CoV-2 and SARS-CoV to invade host cells and tissues (61). CD34+ stem cells, platelets, and MK cell lines express CD13 and EACAM1a (CD66a) (62, 63). Studies have shown that SARS-CoV infects human MK progenitor cells and CD34+ cells (58), exhibiting similar antigenic characteristics to human HCoV-229E (64, 65). HCoV-229E enters monocytes and macrophages via CD13 and CEACAM1a (CD66a) receptors, inducing cell apoptosis (62, 66, 67). Therefore, CD13 and CD66a are potential receptors for viral entry. The interaction between viruses and host cells in the production of specific antibodies (68) can trigger autoimmune antibodies that lead to specific cell death. Consequently, SARS-CoV-2 infection may induce the production of autoantibodies and immune complexes or directly attack hematopoietic stem cells (HSCs) or hematopoietic progenitor cells, resulting in thrombocytopenia. For instance, individuals with thrombocytopenia infected with HIV-1 produce antibodies that cross-react with HIV-1 gp160/120 antigen, leading to increased levels of circulating immune complexes (69, 70). The immune system identifies and targets platelets packaged by antibodies and immune complexes via cells of the reticuloendothelial system, thereby attacking platelets. Hematopoietic cells expressing similar antigens are also vulnerable to damage (63). In summary, viral infections generate antibodies and immune complexes that are recognized by the body’s immune system, causing thrombocytopenia by attacking blood-forming cells. Thus, it is plausible that SARS-CoV-2 might induce the production of autoantibodies and immune complexes or directly target HSCs or hematopoietic progenitor cells, ultimately resulting in thrombocytopenia.

In addition, dysfunction of the local renin-angiotensin system can result in an abnormal bone marrow microenvironment (71, 72). SARS-CoV granules and inflammatory cytokines such as IL-1β and TNF-α promote ACE2 removal from the cell surface. This weakens ACE2 function, leading to renin-angiotensin system dysfunction and increased inflammation (68). Megakaryocytes are also present in the blood vessels of lung tissue, where they contribute to platelet release (73). When the virus attacks lung tissue, it damages lung epithelial cells and endothelial cells, causing vascular leakage and releasing large amounts of pro-inflammatory cytokines and chemokines (68). SARS patients often exhibit diffuse alveolar injury characterized by pulmonary tissue congestion, pulmonary edema, alveolar hyaline membrane formation, and fibrosis (62, 64). Extensive damage to the alveoli leads to the destruction of lung capillaries and subsequent lysis of megakaryocytes, resulting in thrombocytopenia (73, 74). Consequently, elevated levels of chemokines, inflammatory factors, growth factors, and anti-inflammatory factors affect the hematopoietic microenvironment. Moreover, an imbalance in the bone marrow microenvironment may impair thrombopoietin production as well as megakaryocyte differentiation and maturation to some extent, thereby contributing to thrombocytopenia.

### 3.2 Cytokine storm and immune system-mediated thrombocytopenia in COVID-19

During the COVID-19 pandemic, it has been observed that COVID-19 is a highly coagulant disorder associated with inflammation commonly seen in ICU settings. This phenomenon is often referred to as “thromboinflammation” and is linked to cytokine storms (75). In the case of COVID-19, the cytokines involved in cytokine storm mainly include tumor necrosis factor-alpha (TNF-α), interleukin 1β (IL-1β), interleukin 6 (IL-6), interleukin 8 (IL-8), interleukin 10 (IL-10), interferon alpha (IFN-α), and granulocyte-macrophage colony-stimulating factor (GM-CSF) (56). When these cytokines are stimulated and activated, they cause inflammation, damage tissues, and lead to organ dysfunction. Additionally, many cytokines such as IL-3, IL-6, IL-9, IL-1, and stem cell factors can stimulate MK production (76–79). However, tumor growth factor-beta (TGF-β), platelet factor 4, and interferon-alpha (IFN-α) inhibit MK production (80). Elevated TGF-β levels have been observed in SARS patients. Plasma from active SARS patients inhibited the formation of MK colony-forming units, which could be neutralized by anti-TGF-β antibodies. This suggests that virus-induced TGF-β-mediated cytokine storms inhibit megakaryocyte generation, resulting in thrombocytopenia (58). IFN-α induces the production of cytokine signal transduction inhibitors that inhibit the expression of megakaryocyte-regulating transcription factors to some extent. This directly inhibits thrombopoietin (TPO)-mediated MK growth. Ultrastructural research supports this mechanism (81, 82), further indicating insufficient platelet production.

In summary, the specific mechanism of cytokine storm causing thrombocytopenia in COVID-19 is still being investigated. However, it is evident that when infected with COVID-19, a cytokine storm can lead to platelet disorders or thrombocytopenia by either directly destroying platelets or stimulating the production of antibodies that bind and destroy platelets.

Additionally, cases of thrombus formation with thrombocytopenia have been reported following administration of the AstraZeneca recombinant adenovirus vector vaccine (ChAdOx1 nCov-19) (83). This phenomenon is referred to as vaccine-induced immune thrombotic thrombocytopenia (VITT) or thrombosis with thrombocytopenia syndrome (TTS) (84). Studies have shown that some mechanisms of VITT are similar to those of heparin-induced thrombocytopenia (HIT), particularly involving platelet-activating anti-platelet factor 4 (anti-PF4) antibodies (85). Although pathogenic platelet-activating antibodies in VITT caused by vaccination are not common (86), it is important to actively explore the relevant mechanisms and implement effective prevention measures.

In conclusion, there are multiple mechanisms contributing to thrombocytopenia in critically ill patients and those infected with COVID-19, often involving a combination of several factors. It is crucial to consider different pathophysiological mechanisms when treating thrombocytopenia to effectively address the condition.

## 4 Treatments

### 4.1 Treatments of thrombocytopenia in sepsis

In the context of sepsis, several potential therapeutic targets have been identified for managing thrombocytopenia. Interleukin-11 (IL-11) has been shown to effectively prevent and treat chemotherapy-associated thrombocytopenia by increasing the production and differentiation of megakaryocytes. Studies have demonstrated that sepsis patients treated with IL-11 have a lower mortality rate (87). However, the use of IL-11 is limited due to serious side effects and is no longer used in clinical practice.

Another potential treatment option is recombinant human thrombopoietin, which has shown promising results in improving platelet counts more rapidly to normal levels and reducing the need for platelet transfusions in sepsis patients (88).

An animal study revealed that platelet granase B-mediated apoptosis occurs in the spleen and lung during sepsis. The progression of sepsis was found to be slowed down by inhibiting granase B using the platelet GPIIb/IIIa receptor inhibitor etibatitide both *in vitro* and *in vivo*. Inhibitors of GPIIb/IIIa receptors and other antiplatelet drugs delayed survival in mice with sepsis (89).

TAK-242, a compound that binds to TLR4 and inhibits lipopolysaccharide (LPS) activation, has shown potential as a toll-like receptor 4 (TLR4) inhibitor for treating immune factor-induced thrombocytopenia in sepsis (90, 91). Clinical studies using anti-TLR4 antibodies have demonstrated increased survival rates in mice with LPS endotoxemia (92). Additionally, TLR3, TLR5 antagonists, or TLR9 agonists have also improved survival rates in mice with LPS endotoxemia, suggesting their potential use in treating sepsis-associated thrombocytopenia (93, 94).

### 4.2 Treatments of thrombocytopenia in COVID-19

In the context of COVID-19, several treatment options have shown promise for managing thrombocytopenia. Tocilizumab (TCZ), a recombinant humanized monoclonal antibody against the interleukin-6 (IL-6) receptor, has demonstrated significant efficacy in treating cytokine release syndrome in COVID-19 patients. It binds to membranous IL-6R (mIL-6R) and soluble IL-6R (sIL-6R) (95). An Italian study reported that intravenous tocilizumab significantly improved the prognosis of ICU patients (96). Anakinra, an antagonist of IL-1R, has been suggested to block the secretion of IL-1β by macrophages, preventing tissue damage and inhibiting excessive platelet accumulation by blocking endothelial cell exposure and coagulation cascade propagation. Notably, patients treated with IL-1R inhibitors have shown better outcomes compared to those treated with IL-6R inhibitors (97).

Complement inhibitors have also shown potential for managing thrombocytopenia in COVID-19. Inhibition of the complement system can help inhibit abnormal activation of the complement cascade and thrombotic microangiopathies. Studies using eculizumab, a C5-blocking agent, demonstrated a rapid reduction in lactic acid levels, improvement in hypoxia, restoration of platelet counts, and improved prothrombin time (PT) within 15 days (98, 99). AMY-101 has been suggested as another complement inhibitor that follows a similar action to eculizumab in inhibiting hyperinflammatory states (100).

Inhibiting chemokines and chemokine receptors may contribute to the recovery of platelet numbers in COVID-19 patients. The anti-CCR5 antibody leronlimab has shown efficacy in reducing IL-6 levels, restoring T cell populations, reducing inflammatory responses, and indirectly mitigating virus-induced damage (101). Further exploration is needed to determine if leronlimab can effectively treat thrombocytopenia caused by COVID-19.

Thrombopoietin receptor agonists (TPO-RAs) are often used to treat COVID-19-related immune thrombocytopenias (ITPs). However, their use carries a risk of thrombosis (102, 103). Steroids, such as corticosteroids, have been used to treat COVID-19-related ITPs and have shown positive effects in reducing systemic inflammatory response and improving patient outcomes (104). A combination therapy using dexamethasone and intravenous immunoglobulin (IVIG) has demonstrated increased platelet counts within 12 hours after treatment and improved bleeding control and oxygenation in severe COVID-19 patients with ITP (105).

Recombinant human ACE2 (hrsACE2), which binds to viral spike proteins, has been explored as a therapeutic option for preventing tissue damage. Intravenous administration of hrsACE2 for seven days in severe COVID-19 patients has been shown to significantly reduce angiotensin II levels, inhibit IL-6 and IL-8-mediated inflammatory response, alleviate organ damage caused by SARS-CoV-2, and indirectly improve thrombocytopenia (106).

By targeting these receptors and utilizing specific drugs, it is possible to inhibit the process of thrombocytopenia in sepsis and COVID-19. Understanding these mechanisms can aid in the development of effective prevention strategies that improve outcomes for sepsis and COVID-19 patients.

## 5 Conclusion

Thrombocytopenia, characterized by low platelet count, is a common symptom observed in both sepsis and COVID-19 patients. The exact mechanisms underlying thrombocytopenia in these conditions are still being researched, but several potential causes have been identified. In sepsis, cytokine storm plays a significant role in inducing thrombocytopenia. Excessive production of cytokines can directly destroy platelets or stimulate the production of antibodies that bind and destroy platelets. Therapeutic targets for managing sepsis-associated thrombocytopenia include IL-11 and recombinant human thrombopoietin, which have shown promising results in improving platelet counts. Additionally, inhibitors targeting TLR4 and GPIIb/IIIa receptors have demonstrated potential in animal studies. In this context, cytokine release syndrome contributes to thrombocytopenia. Drugs like tocilizumab (TCZ) and anakinra have shown efficacy in managing cytokine release syndrome by targeting IL-6 and IL-1 pathways. Complement inhibitors such as eculizumab have demonstrated positive outcomes in reducing inflammation and improving platelet counts. Chemokine inhibitors and thrombopoietin receptor agonists are also being explored as treatment options. However, it is important to maintain a balanced view when considering these treatments. Some therapeutic options come with limitations or risks. For example, IL-11 has serious side effects that restrict its clinical use, while TPO-RAs carry a risk of thrombosis. Furthermore, the effectiveness of certain drugs like leronlimab in treating thrombocytopenia caused by COVID-19 requires further investigation. And looking ahead, continued research is necessary to gain a comprehensive understanding of the mechanisms underlying thrombocytopenia in sepsis and COVID-19. This will enable the development of more targeted and effective treatments. It is crucial to consider the potential benefits and risks associated with each therapeutic approach, taking into account individual patient characteristics and disease severity. While progress has been made in identifying therapeutic targets for thrombocytopenia in sepsis and COVID-19, further research is needed to optimize treatment strategies. A critical evaluation of available options will help ensure that interventions are balanced, comprehensive, and tailored to the specific needs of patients. Prospective studies should focus on identifying biomarkers for early detection, elucidating the interplay between platelets and immune responses, and evaluating the efficacy of targeted therapies.

## Author contributions

ML and HZ contributed to the conception and design of the study. HC, LF, CX, HH, and TT have conducted a literature review and analysis. JC wrote the first draft of the manuscript. All authors contributed to the article and approved the submitted version.

## References

[1] Gauer RL Braun MM. Thrombocytopenia. Am Family Physician (2012) 85(6):612–22. 22534274

[2] Cecconi M Evans L Levy M Rhodes A. Sepsis and septic shock. Lancet (2018) 392:75–87. doi: 10.1016/S0140-6736(18)30696-2 29937192

[3] Giustozzi M Ehrlinder H Bongiovanni D Borovac JA Guerreiro RA Gasecka A. Coagulopathy and sepsis: Pathophysiology, clinical manifestations, and treatment. Blood Rev (2021) 50:100864. doi: 10.1016/j.blre.2021.100864 34217531

[4] Vardon-Bounes F Ruiz S Gratacap MP Garcia C Payrastre B Minville V. Platelets are critical key players in sepsis. Int J Mol Sci (2019) 20(14):8. doi: 10.3390/ijms20143494 PMC6679237 31315248

[5] Thiery-Antier N Binquet C Vinault S Meziani F Boisrame-Helms J Quenot JP. Is thrombocytopenia an early prognostic marker in septic shock? Crit Care Med (2016) 44:764–72. doi: 10.1097/CCM.0000000000001520 26670473

[6] Schupp T Weidner K Rusnak J Jawhar S Forner J Dulatahu F. Diagnostic and prognostic role of platelets in patients with sepsis and septic shock. Platelets (2023) 34:2131753. doi: 10.1080/09537104.2022.2131753 36484263

[7] Greco E Lupia E Bosco O Vizio B Montrucchio G. Platelets and multi-organ failure in sepsis. Int J Mol Sci (2017) 18(10):5. doi: 10.3390/ijms18102200 29053592 PMC5666881

[8] Guan W Ni Z Hu Y Liang W Ou C He J. China med treatment expert, clinical characteristics of coronavirus disease 2019 in China. New Engl J Med (2020) 382:1708–20. doi: 10.1056/NEJMoa2002032 PMC7092819 32109013

[9] Huang C Wang Y Li X Ren L Zhao J Hu Y. Clinical features of patients infected with 2019 novel coronavirus in Wuhan, China. Lancet (2020) 395:497–506. doi: 10.1016/S0140-6736(20)30183-5 31986264 PMC7159299

[10] Rampotas A Pavord S. Platelet aggregates, a marker of severe COVID-19 disease. J Clin Pathol (2021) 74:750–1. doi: 10.1136/jclinpath-2020-206933 33067181

[11] Comer SP Cullivan S Szklanna PB Weiss L Cullen S Kelliher S. COVID-19 induces a hyperactive phenotype in circulating platelets. PloS Biol (2021) 19(2):5. doi: 10.1371/journal.pbio.3001109 PMC7920383 33596198

[12] Parlato M Cavaillon JM. Host response biomarkers in the diagnosis of sepsis: a general overview. Methods Mol Biol (2015) 1237:149–211. doi: 10.1007/978-1-4939-1776-1_15 25319788

[13] Cognasse F Hamzeh H Chavarin P Acquart S Genin C Garraud O. Evidence of Toll-like receptor molecules on human platelets. Immunol Cell Biol (2005) 83:196–8. doi: 10.1111/j.1440-1711.2005.01314.x 15748217

[14] Andonegui G Kerfoot SM McNagny K Ebbert KVJ Patel KD Kubes P. Platelets express functional Toll-like receptor-4. Blood (2005) 106:2417–23. doi: 10.1182/blood-2005-03-0916 15961512

[15] Stéphan F CM Kaplan C Maillet J Novara A Fagon J Bonnet F. Autoantibodies against platelet glycoproteins in critically ill patients with thrombocytopenia. Am J Med (2000) 108(7):554–60. doi: 10.1016/s0002-9343(00)00332-6 10806284

[16] Bedet A Razazi K Boissier F Surenaud M Hue S Giraudier S. Mechanisms of thrombocytopenia during septic shock: A multiplex cluster analysis of endogenous sepsis mediators. Shock (2018) 49:641–8. doi: 10.1097/SHK.0000000000001015 29028771

[17] Claushuis TA van Vught LA Scicluna BP Wiewel MA Klein Klouwenberg PM Hoogendijk AJ. Risk Stratification of Sepsis, Thrombocytopenia is associated with a dysregulated host response in critically ill sepsis patients. Blood (2016) 127:3062–72. doi: 10.1182/blood-2015-11-680744 26956172

[18] Pierrakos C Vincent JL. Sepsis biomarkers: a review. Crit Care (2010) 14:9–11. doi: 10.1186/cc8872 PMC2875530 20144219

[19] Huizinga EG Tsuji S Romijn RAP Schiphorst ME de Groot PG Sixma JJ. Structures of glycoprotein Ib alpha and its complex with von Willebrand factor A1 domain. Science (2002) 297:1176–9. doi: 10.1126/science.107355 12183630

[20] Pu Q Wiel E Corseaux D Bordet R Azrin MA Ezekowitz MD. Beneficial effect of glycoprotein IIb/IIIa inhibitor (AZ-1) on endothelium in Escherichia coli endotoxin-induced shock. Crit Care Med (2001) 29:1181–8. doi: 10.1097/00003246-200106000-00019 11395599

[21] Kumar H Kawai T Akira S. Toll-like receptors and innate immunity. Biochem Biophys Res Commun (2009) 388:621–5. doi: 10.1016/j.bbrc.2009.08.062 19686699

[22] Zhang GY Han JY Welch EJ Ye RD Voyno-Yasenetskaya TA Malik AB. Lipopolysaccharide Stimulates Platelet Secretion and Potentiates Platelet Aggregation via TLR4/MyD88 and the cGMP-Dependent Protein Kinase Pathway. J Immunol (2009) 182:7997–8004. doi: 10.4049/jimmunol.0802884 19494325 PMC2787095

[23] Larkin CM Hante NK Breen EP Tomaszewski KA Eisele S Radomski MW. Role of matrix metalloproteinases 2 and 9, toll-like receptor 4 and platelet-leukocyte aggregate formation in sepsis-associated thrombocytopenia. PloS One (2018) 13:e0196478. doi: 10.1371/journal.pone.0196478 29734352 PMC5937753

[24] Zufferey A Kapur R Semple JW. Pathogenesis and therapeutic mechanisms in immune thrombocytopenia (ITP). J Clin Med (2017) 6(2):3–4. doi: 10.3390/jcm6020016 PMC5332920 28208757

[25] Wang W Irani RA Zhang YJ Ramin SM Blackwell SC Tao LJ. Autoantibody-mediated complement C3a receptor activation contributes to the pathogenesis of preeclampsia. Hypertension (2012) 60:712. doi: 10.1161/HYPERTENSIONAHA.112.191817 22868393 PMC4131740

[26] Li J van der Wal DE Zhu G Xu M Yougbare I Ma L. Desialylation is a mechanism of Fc-independent platelet clearance and a therapeutic target in immune thrombocytopenia. Nat Commun (2015) 6:2. doi: 10.1038/ncomms8737 PMC4518313 26185093

[27] Li J Callum JL Lin YL Zhou Y Zhu GH Ni HY. Severe platelet desialylation in a patient with glycoprotein Ib/IX antibody-mediated immune thrombocytopenia and fatal pulmonary hemorrhage. Haematologica (2014) 99:E61–3. doi: 10.3324/haematol.2013.102897 PMC3971097 24532041

[28] Grozovsky R Begonja AJ Liu K Visner G Hartwig JH Falai H. The Ashwell-Morell receptor regulates hepatic thrombopoietin production via JAK2-STAT3 signaling. Nat Med (2015) 21:47–54. doi: 10.1038/nm.3770 25485912 PMC4303234

[29] Deppermann C Kratofil RM Peiseler M David BA Zindel J Castanheira F. Macrophage galactose lectin is critical for Kupffer cells to clear aged platelets. J Exp Med (2020) 217(4):2. doi: 10.1084/jem.20190723 PMC7144524 31978220

[30] van Velzen JF Laros-van Gorkom BAP Pop GAM van Heerde WL. Multicolor flow cytometry for evaluation of platelet surface antigens and activation markers. Thromb Res (2012) 130:92–8. doi: 10.1016/j.thromres.2012.02.041 22424855

[31] Salat A Bodingbauer G Boehm D Murabito M Tochkow E Sautner T. Changes of platelet surface antigens in patients suffering from abdominal septic shock. Thromb Res (1999) 95:289–94. doi: 10.1016/S0049-3848(99)00046-8 10527406

[32] Fahey VA. Heparin-induced thrombocytopenia. J Vasc Nurs Off Publ Soc Peripheral Vasc Nurs (1995) 13:112–6. doi: 10.1016/S1062-0303(05)80003-2 8703791

[33] Perdomo J Leung HHL Ahmadi Z Feng Y Passam FH Chong BH. Neutrophil activation and netosis are the key drivers of thrombosis in heparin-induced thrombocytopenia. Blood (2018) 132:378–8. doi: 10.1182/blood-2018-99-116421 PMC6428879 30899022

[34] Warkentin TE. HIT: still stringing us along. Blood (2020) 135:1193–4. doi: 10.1182/blood.2020005157 32271906

[35] Masucci MT Minopoli M Del Vecchio S Carriero MV. The emerging role of neutrophil extracellular traps (NETs) in tumor progression and metastasis. Front Immunol (2020) 11:1749. doi: 10.3389/fimmu.2020.01749 33042107 PMC7524869

[36] Wigerblad G Kaplan MJ. Neutrophil extracellular traps in systemic autoimmune and autoinflammatory diseases. Nat Rev Immunol (2022) 23(5):274–88. doi: 10.1038/s41577-022-00787-0 PMC9579530 36257987

[37] Etulain J Martinod K Wong SL Cifuni SM Schattner M Wagner DD. P-selectin promotes neutrophil extracellular trap formation in mice. Blood (2015) 126:242–6. doi: 10.1182/blood-2015-01-624023 PMC4497964 25979951

[38] Wang H Wang C Zhao MH Chen M. Neutrophil extracellular traps can activate alternative complement pathways. Clin Exp Immunol (2015) 181:518–27. doi: 10.1111/cei.12654 PMC4557387 25963026

[39] Crow AR Song S Semple JW Freedman J Lazarus AH. IVIg inhibits reticuloendothelial system function and ameliorates murine passive-immune thrombocytopenia independent of anti-idiotype reactivity. Br J Haematology (2001) 115:679–86. doi: 10.1046/j.1365-2141.2001.03136.x 11736954

[40] Samuelsson A Towers TL Ravetch JV. Anti-inflammatory activity of IVIG mediated through the inhibitory Fc receptor. Science (2001) 291:484–6. doi: 10.1126/science.291.5503.484 11161202

[41] Francois B Trimoreau F Vignon P Fixe P Praloran V Gastinne H. Thrombocytopenia in the sepsis syndrome: Role of hemophagocytosis and macrophage colony-stimulating factor. Am J Med (1997) 103:114–20. doi: 10.1016/S0002-9343(97)00136-8 9274894

[42] Bibas M. Bleeding and coagulopathies in critical care. New Engl J Med (2014) 370:2152–2. doi: 10.1056/NEJMc1403768 24869735

[43] Johansson D Shannon O Rasmussen M. Platelet and neutrophil responses to gram positive pathogens in patients with bacteremic infection. PloS One (2011) 6(11):4–5. doi: 10.1371/journal.pone.0026928 PMC3226579 22140434

[44] Hayakawa M Sawamura A Gando S Jesmin S Naito S Ieko M. and insufficient activation of fibrinolysis by both plasmin and neutrophil elastase promote organ dysfunction in disseminated intravascular coagulation associated with sepsis. Thromb Res (2012) 130:906–13. doi: 10.1016/j.thromres.2012.01.015 22353215

[45] Gando S. Role of fibrinolysis in sepsis. Semin Thromb Hemostasis (2013) 39:392–9. doi: 10.1055/s-0033-1334140 23446914

[46] Levi M van der Poll T Buller HR. Bidirectional relation between inflammation and coagulation. Circulation (2004) 109:2698–704. doi: 10.1161/01.CIR.0000131660.51520.9A 15184294

[47] Foley JH Conway EM. Cross talk pathways between coagulation and inflammation. Circ Res (2016) 118:1392–408. doi: 10.1161/CIRCRESAHA.116.306853 27126649

[48] Lupu F Keshari RS Lambris JD Coggeshall KM. Crosstalk between the coagulation and complement systems in sepsis. Thromb Res (2014) 133:S28–31. doi: 10.1016/j.thromres.2014.03.014 PMC4154483 24759136

[49] Iba T Levy JH Raj A Warkentin TE. Advance in the management of sepsis-induced coagulopathy and disseminated intravascular coagulation. J Clin Med (2019) 8(5):2–5. doi: 10.3390/jcm8050728 PMC6572234 31121897

[50] Kinasewitz GT Yan SB Basson B Comp P Russell JA Cariou A. Universal changes in biomarkers of coagulation and inflammation occur in patients with severe sepsis, regardless of causative micro-organism ISRCTN74215569. Crit Care (2004) 8:R82–90. doi: 10.1186/cc2459 PMC420030 15025782

[51] Fakhouri F Zuber J Fremeaux-Bacchi V Loirat C. Haemolytic uraemic syndrome. Lancet (2017) 390:681–96. doi: 10.1016/S0140-6736(17)30062-4 28242109

[52] Lee KH Hui KP Tan WC. Thrombocytopenia in sepsis: a predictor of mortality in the intensive care unit. Singapore Med J (1993) 34(3):245–6. 8266183

[53] Stephan F Hollande J Richard O Cheffi A Maier-Redelsperger M Flahault A. Thrombocytopenia in a surgical ICU. Chest (1999) 115:1363–70. doi: 10.1378/chest.115.5.1363 10334154

[54] Al-Tawfiq JA Hinedi K Abbasi S Babiker M Sunji A Eltigani M. Hematologic, hepatic, and renal function changes in hospitalized patients with Middle East respiratory syndrome coronavirus. Int J Lab Hematol (2017) 39:272–8. doi: 10.1111/ijlh.12620 PMC7165514 28444873

[55] Lippi G Plebani M Henry BM. Thrombocytopenia is associated with severe coronavirus disease 2019 (COVID-19) infections: A meta-analysis. Clin Chim Acta (2020) 506:145–8. doi: 10.1016/j.cca.2020.03.022 PMC7102663 32178975

[56] Ragab D Salah Eldin H Taeimah M Khattab R Salem R. The COVID-19 cytokine storm; what we know so far. Front Immunol (2020) 11:1446. doi: 10.3389/fimmu.2020.01446 32612617 PMC7308649

[57] Yang M Ng MH Li CK. Thrombocytopenia in patients with severe acute respiratory syndrome (review). Hematol (Amsterdam Netherlands) (2005) 10:101–5. doi: 10.1080/10245330400026170 16019455

[58] Yang M Ng MH Li CK Chan PK Liu C Ye JY. Thrombopoietin levels increased in patients with severe acute respiratory syndrome. Thromb Res (2008) 122:473–7. doi: 10.1016/j.thromres.2007.12.021 PMC7112012 18314161

[59] Drissen R Buza-Vidas N Woll P Thongjuea S Gambardella A Giustacchini A. Distinct myeloid progenitor-differentiation pathways identified through single-cell RNA sequencing. Nat Immunol (2016) 17:666. doi: 10.1038/ni.3412 27043410 PMC4972405

[60] Kaushansky K. Historical review: megakaryopoiesis and thrombopoiesis. Blood (2008) 111:981–6. doi: 10.1182/blood-2007-05-088500 PMC2214745 18223171

[61] Mercurio I Tragni V Busto F De Grassi A Pierri CL. Protein structure analysis of the interactions between SARS-CoV-2 spike protein and the human ACE2 receptor: from conformational changes to novel neutralizing antibodies. Cell Mol Life Sci (2021) 78:1501–22. doi: 10.1007/s00018-020-03580-1 PMC7334636 32623480

[62] Yang M Li CK Li K Hon KLE Ng MHL Chan PKS. Hematological findings in SARS patients and possible mechanisms (Review). Int J Mol Med (2004) 14:311–5. doi: 10.3892/ijmm.14.2.311 15254784

[63] Jeffers SA Tusell SM Gillim-Ross L Hemmila EM Achenbach JE Babcock GJ. CD209L (L-SIGN) is a receptor for severe acute respiratory syndrome coronavirus. Proc Natl Acad Sci United States America (2004) 101:15748–53. doi: 10.1073/pnas.0403812101 PMC524836 15496474

[64] Peiris JSM Lai ST Poon LLM Guan Y Yam LYC Lim W. Coronavirus as a possible cause of severe acute respiratory syndrome. Lancet (2003) 361:1319–25. doi: 10.1016/S0140-6736(03)13077-2 PMC7112372 12711465

[65] Yu CJ Chen YC Hsiao CH Kuo TC Chang SC Lu CY. Identification of a novel protein 3a from severe acute respiratory syndrome coronavirus. FEBS Lett (2004) 565:111–6. doi: 10.1016/j.febslet.2004.03.086 PMC7126674 15135062

[66] Nomura R Kiyota A Suzaki E Kataoka K Senda T Fujimoto T. Human coronavirus 229E binds to CD13 in raft and enters the cell through caveolae. Anatomical Sci Int (2004) 79:287. doi: 10.1128/JVI.78.16.8701-8708.2004 PMC479086 15280478

[67] Hammarstrom S. The carcinoembryonic antigen (CEA) family: structures, suggested functions and expression in normal and Malignant tissues. Semin Cancer Biol (1999) 9:67–81. doi: 10.1006/scbi.1998.0119 10202129

[68] Fu YJ Cheng YX Wu YT. Understanding SARS-coV-2-mediated inflammatory responses: from mechanisms to potential therapeutic tools. Virologica Sin (2020) 35:266–71. doi: 10.1007/s12250-020-00207-4 PMC7090474 32125642

[69] Scaradavou A. HIV-related thrombocytopenia. Blood Rev (2002) 16:73–6. doi: 10.1054/blre.2001.0188 11914001

[70] Nardi M Tomlinson S Greco MA Karpatkin S. Complement-independent, peroxide-induced antibody lysis of platelets in HIV-1-related immune thrombocytopenia. Cell (2001) 106:551–61. doi: 10.1016/S0092-8674(01)00477-9 11551503

[71] Strawn WB Richmond RS Tallant EA Gallagher PE Ferrario CM. Renin-angiotensin system expression in rat bone marrow haematopoietic and stromal cells. Br J Haematology (2004) 126:120–6. doi: 10.1111/j.1365-2141.2004.04998.x 15198742

[72] Haznedaroglu IC Malkan UY. Local bone marrow renin-angiotensin system in the genesis of leukemia and other malignancies. Eur Rev Med Pharmacol Sci (2016) 20(19):4089–111. 27775788

[73] Lefrancais E Ortiz-Munoz G Caudrillier A Mallavia B Liu F Sayah DM. The lung is a site of platelet biogenesis and a reservoir for haematopoietic progenitors. Nature (2017) 544:105–9. doi: 10.1038/nature21706 PMC5663284 28329764

[74] Weibel ER. Oxygen effect on lung cells. Arch Internal Med (1971) 128:54–6. doi: 10.1001/archinte.1971.00310190058005 4932533

[75] Bikdeli B Madhavan MV Gupta A Jimenez D Burton JR Nigoghossian CD. Pharmacological agents targeting thromboinflammation in COVID-19: review and implications for future research. Thromb Haemostasis (2020) 120:1004–24. doi: 10.1055/s-0040-1713152 PMC7516364 32473596

[76] Sugimoto N Eto K. Platelet production from induced pluripotent stem cells. J Thromb Haemost (2017) 15:1717–27. doi: 10.1111/jth.13736 28752663

[77] Monroy RL Davis TA Donahue RE MacVittie TJ. *In vivo* stimulation of platelet production in a primate model using IL-1 and IL-3. Exp Hematol (1991) 19(7):629–35. 1893949

[78] Williams N Bertoncello I Jackson H Arnold J Kavnoudias H. The role of interleukin 6 in megakaryocyte formation, megakaryocyte development and platelet production. Ciba Foundation symposium (1992) 167:160–70; discussion 170-3. doi: 10.1002/9780470514269.ch10 1425011

[79] Cortin V Garnier A Pineault N Lemieux R Boyer L Proulx C. Efficient *in vitro* megakaryocyte maturation using cytokine cocktails optimized by statistical experimental design. Exp Hematol (2005) 33:1182–91. doi: 10.1016/j.exphem.2005.06.020 16219540

[80] Behrens K Alexander WS. Cytokine control of megakaryopoiesis. Growth Factors (2018) 36:89–103. doi: 10.1080/08977194.2018.1498487 30318940

[81] Yamane A Nakamura T Suzuki H Ito M Ohnishi Y Ikeda Y. Interferon-alpha 2b-induced thrombocytopenia is caused by inhibition of platelet production but not proliferation and endomitosis in human megakaryocytes. Blood (2008) 112:542–50. doi: 10.1182/blood-2007-12-125906 18523149

[82] Wang Q Fox NE Kaushansky K. Interferon-alpha directly inhibits thrombopoietin-induced megakaryocyte proliferation and differentiation. Zhonghua Xue Ye Xue Za Zhi (2001) 22(6):296–9. 11877087

[83] Sadoff J Davis K Douoguih M. Thrombotic thrombocytopenia after ad26.COV2.S vaccination - response from the manufacturer. New Engl J Med (2021) 384:1965–6. doi: 10.1056/NEJMc2106075 PMC8117965 33861522

[84] Elsheikh S Lip GYH. Editorial commentary: COVID-19 and COVID-19 vaccination: Observations on thrombosis and thrombocytopenia. Trends Cardiovasc Med (2022) 32:257–8. doi: 10.1016/j.tcm.2022.03.002 PMC8916829 35288299

[85] Iba T Levy JH. Thrombosis and thrombocytopenia in COVID-19 and after COVID-19 vaccination. Trends Cardiovasc Med (2022) 32:249–56. doi: 10.1016/j.tcm.2022.02.008 PMC8861143 35202800

[86] Thiele T Ulm L Holtfreter S Schonborn L Kuhn SO Scheer C. Frequency of positive anti-PF4/polyanion antibody tests after COVID-19 vaccination with ChAdOx1 nCoV-19 and BNT162b2. Blood (2021) 138:299–303. doi: 10.1182/blood.2021012217 33988688 PMC8129797

[87] Wan B Zhang H Fu HY Chen YK Yang LP Yin JT. Recombinant human interleukin-11 (IL-11) is a protective factor in severe sepsis with thrombocytopenia: A case-control study. Cytokine (2015) 76:138–43. doi: 10.1016/j.cyto.2015.08.001 26276375

[88] Wu Q Ren JA Wu XW Wang GF Gu GS Liu S. Recombinant human thrombopoietin improves platelet counts and reduces platelet transfusion possibility among patients with severe sepsis and thrombocytopenia: A prospective study. J Crit Care (2014) 29:362–6. doi: 10.1016/j.jcrc.2013.11.023 24405656

[89] Sharron M Hoptay CE Wiles AA Garvin LM Geha M Benton AS. Platelets induce apoptosis during sepsis in a contact-dependent manner that is inhibited by GPIIb/IIIa blockade. PloS One (2012) 7(7):6. doi: 10.1371/journal.pone.0041549 PMC3406039 22844498

[90] Samarpita S Kim JY Rasool MK Kim KS. Investigation of toll-like receptor (TLR) 4 inhibitor TAK-242 as a new potential anti-rheumatoid arthritis drug. Arthritis Res Ther (2020) 22(1):8–9. doi: 10.1186/s13075-020-2097-2 PMC6979396 31973752

[91] Wittebole X Castanares-Zapatero D Laterre PF. Toll-like receptor 4 modulation as a strategy to treat sepsis. Mediators Inflammation (2010) 2010:3–4. doi: 10.1155/2010/568396 PMC2855078 20396414

[92] Roger T Froidevaux C Le Roy D Reymond MK Chanson AL Mauri D. Protection from lethal Gram-negative bacterial sepsis by targeting Toll-like receptor 4. Proc Natl Acad Sci United States America (2009) 106:2348–52. doi: 10.1073/pnas.0808146106 PMC2650125 19181857

[93] Ranjith-Kumar CT Duffy KE Jordan JL Eaton-Bassiri A Vaughan R Hoose SA. Single-stranded oligonucleotides can inhibit cytokine production induced by human toll-like receptor 3. Mol Cell Biol (2008) 28:4507–19. doi: 10.1128/MCB.00308-08 PMC2447120 18490443

[94] Lahiri A Lahiri A Das P Vani J Shaila MS Chakravortty D. TLR 9 activation in dendritic cells enhances salmonella killing and antigen presentation via involvement of the reactive oxygen species. PloS One (2010) 5(10):2–3. doi: 10.1371/journal.pone.0013772 PMC2966436 21048937

[95] Zhang S Li L Shen A Chen Y Qi Z. Rational use of tocilizumab in the treatment of novel coronavirus pneumonia. Clin Drug Invest (2020) 40:511–8. doi: 10.1007/s40261-020-00917-3 PMC7183818 32337664

[96] Toniati P Piva S Cattalini M Garrafa E Regola F Castelli F. Tocilizumab for the treatment of severe COVID-19 pneumonia with hyperinflammatory syndrome and acute respiratory failure: A single center study of 100 patients in Brescia, Italy. Autoimmun Rev (2020) 19(7):4. doi: 10.1016/j.autrev.2020.102568 PMC7252115 32376398

[97] Nemchand P Tahir H Mediwake R Lee J. Cytokine storm and use of anakinra in a patient with COVID-19. BMJ Case Rep (2020) 13(9):2. doi: 10.1136/bcr-2020-237525 PMC7493115 32933914

[98] Annane D Heming N Grimaldi-Bensouda L Fremeaux-Bacchi V Vigan M Roux A-L. Eculizumab as an emergency treatment for adult patients with severe COVID-19 in the intensive care unit: A proof-of-concept study. Eclinicalmedicine (2020) 28:4–5. doi: 10.1016/j.eclinm.2020.100590 PMC7644240 33173853

[99] Mastaglio S Ruggeri A Risitano AM Angelillo P Yancopoulou D Mastellos DC. The first case of COVID-19 treated with the complement C3 inhibitor AMY-101. Clin Immunol (2020) 215:3. doi: 10.1016/j.clim.2020.108450 PMC7189192 32360516

[100] Mastellos DC da Silva B Fonseca BAL Fonseca NP Auxiliadora-Martins M Mastaglio S. Complement C3 vs C5 inhibition in severe COVID-19: Early clinical findings reveal differential biological efficacy. Clin Immunol (2020) 220:8. doi: 10.1016/j.clim.2020.108598 PMC7501834 32961333

[101] Patterson BK Seethamraju H Dhody K Corley MJ Kazempour K Lalezari J. CCR5 inhibition in critical COVID-19 patients decreases inflammatory cytokines, increases CD8 T-cells, and decreases SARS-CoV2 RNA in plasma by day 14. Int J Infect Dis (2021) 103:25–32. doi: 10.1016/j.ijid.2020.10.101 33186704 PMC7654230

[102] Siegal D Crowther M Cuker A. Thrombopoietin receptor agonists in primary immune thrombocytopenia. Semin Hematol (2013) 50(Suppl 1):S18–21. doi: 10.1053/j.seminhematol.2013.03.005 PMC3658154 23664510

[103] Alonso-Beato R Morales-Ortega A Fernandez F Moron AIP Rios-Fernandez R Rubio JLC. Immune thrombocytopenia and COVID-19: Case report and review of literature. Lupus (2021) 30:1515–21. doi: 10.1177/09612033211021161 34053365

[104] Albani F Fusina F Granato E Capotosto C Ceracchi C Gargaruti R. Corticosteroid treatment has no effect on hospital mortality in COVID-19 patients. Sci Rep (2021) 11:2–4. doi: 10.1038/s41598-020-80654-x PMC7806743 33441909

[105] Martincic Z Skopec B Rener K Mavric M Vovko T Jereb M. Severe immune thrombocytopenia in a critically ill COVID-19 patient. Int J Infect Dis (2020) 99:269–71. doi: 10.1016/j.ijid.2020.08.002 PMC7409801 32771636

[106] Zoufaly A Poglitsch M Aberle JH. Human recombinant soluble ACE2 in severe COVID-19. Lancet Respir Med (2020) 8:E78–8. doi: 10.1016/S2213-2600(20)30418-5 PMC7515587 33131609

